# Genetic Susceptibility to Fungal Infections and Links to Human Ancestry

**DOI:** 10.3389/fgene.2021.709315

**Published:** 2021-08-19

**Authors:** Bharati Naik, Sumayyah M. Q. Ahmed, Suparna Laha, Shankar Prasad Das

**Affiliations:** Yenepoya Research Centre, Yenepoya (Deemed to be University), Mangalore, India

**Keywords:** genetic predisposition, disease susceptibility, invasive, fungal infection, host genetics, genetic polymorphism, SNP, human ancestry

## Abstract

Over the ages, fungi have associated with different parts of the human body and established symbiotic associations with their host. They are mostly commensal unless there are certain not so well-defined factors that trigger the conversion to a pathogenic state. Some of the factors that induce such transition can be dependent on the fungal species, environment, immunological status of the individual, and most importantly host genetics. In this review, we discuss the different aspects of how host genetics play a role in fungal infection since mutations in several genes make hosts susceptible to such infections. We evaluate how mutations modulate the key recognition between the pathogen associated molecular patterns (PAMP) and the host pattern recognition receptor (PRR) molecules. We discuss the polymorphisms in the genes of the immune system, the way it contributes toward some common fungal infections, and highlight how the immunological status of the host determines fungal recognition and cross-reactivity of some fungal antigens against human proteins that mimic them. We highlight the importance of single nucleotide polymorphisms (SNPs) that are associated with several of the receptor coding genes and discuss how it affects the signaling cascade post-infection, immune evasion, and autoimmune disorders. As part of personalized medicine, we need the application of next-generation techniques as a feasible option to incorporate an individual’s susceptibility toward invasive fungal infections based on predisposing factors. Finally, we discuss the importance of studying genomic ancestry and reveal how genetic differences between the human race are linked to variation in fungal disease susceptibility.

## Introduction

**FIGURE 1:**
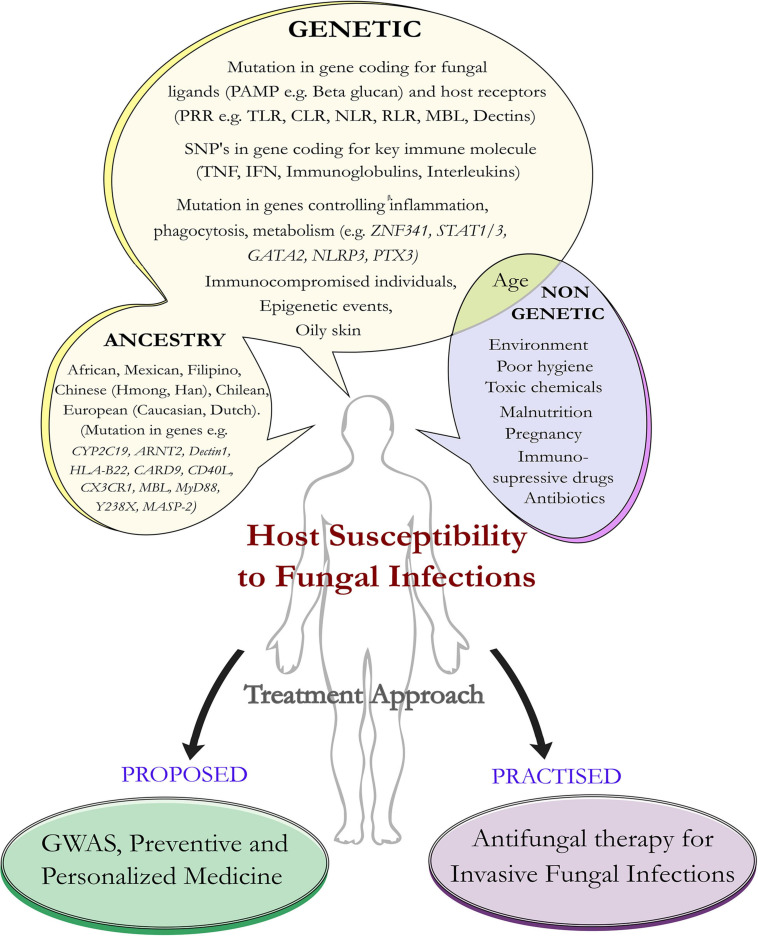
Host susceptibility to invasive fungal infection: predisposing factors and treatment approach. Schematic diagram represents predisposition of the host to certain factors that make them susceptible to fungal infections. Such factors can be genetic as well as non-genetic. Apart from genetic mutations in the host ligand, fungal receptors, and immune genes, human ancestry plays an important role in susceptibility toward invasive fungal infections. The future approaches would be geared toward the investigation (as part of preventive medicine) of the genetic mutations that predispose individuals to fungal infections and offer personalized medicine compared to the more traditional approach that is practiced in the form of antifungal medication.

Fungi are eukaryotic organisms that have a tremendous impact on human health. About 5.1 million fungal species are present on the earth (Hawksworth and Rossman, 1997; Blackwell, 2011). They reproduce asexually by sporulation, budding, and fragmentation. Sexual reproduction involves three phases like plasmogamy, karyogamy, and meiosis. In fungi, hyphae are the main mode of vegetative growth and are collectively called the mycelium. They are usually heterotrophic in nature (Carris et al., 2012) and few are commensal, with the human body acting as a host (Ibrahim and Voelz, 2017). Most of the fungi are adapted to the land environments, and during early episodes of terrestrialization, they had interacted with other organisms having typical parasitic lifestyles (Naranjo-Ortiz and Gabaldón, 2019). Under certain not so well-defined conditions, fungi transform from the non-pathogenic budding yeast to its pathogenic hyphal form, which invades the host tissue (de Pauw, 2011; Underhill and Pearlman, 2015; Kruger et al., 2019; Rai et al., 2021). The fungal species can grow anywhere including plants, animals, and humans. Some enters into our body by inhalation (e.g., *Aspergillus*) and some are commensal (e.g., *Candida*, *Malassezia*) (Underhill and Pearlman, 2015). Commensal like *Malassezia* is more abundant in sebaceous sites of the host. Since they are lipid dependent, they obtain food sources from the host without harming them and colonization starts immediately after birth, when neonatal sebaceous glands are active (Vijaya Chandra et al., 2021). Studies of the microbiome have emerged to be an important area of research, and more importantly, the spotlight is now to understand less studied fungi that have a tremendous influence on the human microbiome especially among immunocompromised individuals. A dysbiotic microbial population is a general characteristic of any fungal infection affecting the mammalian system (Iliev and Leonardi, 2017). Recent reports point toward the role of fungus in pancreatic ductal adenocarcinoma (PDA), a form of human pancreatic cancer caused directly by the presence of budding yeast *Malassezia*, which colonizes the human gut (Aykut et al., 2019). The severity of fungal infection depends on factors such as inoculum load, magnitude of tissue destruction, ability of the fungus to multiply in the tissue, ability to migrate to nearby organs, microenvironment, and immunogenetic status of the host. Resistance to fungi externally is based on cutaneous and mucosal physical barriers and internally by the body’s immune molecules and the defensins (Aristizabal and González, 2013; Coates et al., 2018; Salazar and Brown, 2018). Immunosuppression and breakdown of anatomical barriers such as the skin are the major factors behind fungal infections (Kobayashi, 1996). In addition to this, malnutrition, poor hygiene, use of antibiotics, genetic predisposition, environmental factors, and host physiological factors (e.g., oily skin) contribute toward disease progression (Figure 1).

Genetic variations play an important role in fungal infection (Pana et al., 2014; Maskarinec et al., 2016; Duxbury et al., 2019; Table 1). Recent studies have shown the importance of host genetic variation in influencing the severity and susceptibility to invasive fungal infections (IFIs) (Maskarinec et al., 2016). Increased incidence of opportunistic fungal diseases has been implicated due to gene polymorphism, and genetic errors are frequently observed in immunodeficient phenotypes (Pana et al., 2014). Along with genetic and environmental factors, lifestyle also contributes toward the variation in the genome, as the presence of toxic chemicals and immunosuppressive drugs in an organism’s environment leads to altered immune status and inherited deficiencies, which result in susceptibility toward fungal infection (Kumar et al., 2018; Figure 1). At the molecular level, epigenetic events like alteration of DNA methylation (a key feature that controls gene expression) (Martin and Fry, 2018), modification in the histones (involved in altered gene expression) (Dolinoy and Jirtle, 2008), and interaction between microbes, genotypes, and environment play a key role in disease progression (Goodrich et al., 2017). Now, challenge for biologists is to identify genetic components that predispose individuals to fungal infection. The study of genes will help to understand the relationship between genetic polymorphism and the cellular phenotype of host and pathogen (Sardinha et al., 2011). Recent research outcomes aided by genomic sequencing point toward an interesting link between genetic predisposition to fungal infections and human ancestry. Single nucleotide polymorphism (SNP) in key immune genes plays an important role in fungal infection affecting particular ancestral populations (Hughes et al., 2008; Dominguez-Andres and Netea, 2019). Thus, with the availability of genetic information, we can study the mechanism behind host defense against the pathogen, susceptibility toward infection (Sardinha et al., 2011), and also have an idea of how the pathogens are evolving and trying to adapt to their host environment through host-pathogen interactions.

**TABLE 1:** Genetic mutations, human ancestry, and fungal infections.

**Immune response**		**Genes**	Ancestry link**	**Fungal pathogen**	**Diseases**
Innate immunity	Cell mediated	DOCK8, MyD88**, CARD9**, NCF1, TLR1, MPO, CYBB, CYBA, NADPH oxidase	Chinese (Han) African	*Candida*	Chronic mucocutaneous candidiasis (CMC), chronic granulomatous disease (CGD), candidemia
		*PTX3, NCF1, NCF2, NCF4, DOCK8, TLR4, NADPH oxidase, MPO*		*Aspergillus*	Invasive aspergillosis (IA)
		*CARD9, DOCK8*		*Malassezia*	Pityriasis versicolor
		MBL, MASP-2**	Chinese	*Sporothrix*	Sporotrichosis
		MBL**	Chinese	*Pneumocystis jirovecii*	Pneumocystis pneumonia (PCP)
				*Candida*	Recurrent vulvovaginal candidiasis (RVVC)
		*Ferroxidase*		Mucorales	Mucormycosis
		HLA-B22**	Mexican	*Histoplasma capsulatum*	Histoplasmosis
	Humoral	IL-17F, Act1, IL-12RB1, IL-17R, IL-17A, IL-17RA, IL-4, IL-12, TyK2, IL-17RC ZNF341, IL-17, IL-22, *Y238X, CLEC7A*, IL-10		*Candida, Histoplasma capsulatum*	Chronic mucocutaneous candidiasis (CMC), recurrent vulvovaginal candidiasis (RVVC), histoplasmosis, hyper–immunoglobulin E syndrome (HIES)
		Dectin 1**, IL-10	Chinese (Han) Dutch	*Coccidioides immitis*	Coccidioidomycosis
		Y238X**, IL-10**, TNFα**, IFN-γ**, CLEC7A, CX3CR1**, ARNT2** Asp299Gly, Thr39lle	European (Dutch, Caucasian)	*Aspergillus*	Invasive pulmonary aspergillosis (IPA)
		*rs2243250(IL-4)*		*Pneumocystis jirovecii*	Pneumocystis pneumonia (PCP)
		IL-6		*Blastomyces*	Blastomycosis
		IL-2		*Histoplasma*	Histoplasmosis
Adaptive immunity	Cell mediated	*STAT1*		*Histoplasma*	Histoplasmosis
				*Coccidiodes*	Coccidioidomycosis
		STAT1, STAT3, AIRE, GATA2, RORC, CYP2C19**, RAG1, RAG2	Chinese (Han) Chilean	*Candida*	Candidiasis
		NOD2, STAT3, CYP2C19**	Chinese (Han)	*Aspergillus*	Aspergillosis
		CD40L ** CD50, CD80	Chinese mainland	*Pneumocystis jirovecii, Trichophyton*	Invasive fungal infection (IFI)
	Humoral	IgG, IgA, IgE, IgM, defect in MHC class II molecule		*Pneumocystis jirovecii Candida, Aspergillus, Blastomyces, Coccidioides, Cryptococcus, Histoplasma, Paracoccidioides*	Pneumocystis pneumonia (PCP), candidiasis, aspergillosis, blastomycosis, coccidioidomycosis, cryptococcosis, histoplasmosis, paracoccidioidomycosis.

The symbol ** is used for the genes having the ancestry link.

## Genetic Predisposition and Host-Pathogen Interaction

An opportunistic fungus causes diseases mostly in immunocompromised individuals, though normal individuals are also affected (Low and Rotstein, 2011; Eades and Armstrong-James, 2019). Host-pathogen communication initiates through the interactions of the fungal ligands and receptors present on the skin and internal organs (Richmond and Harris, 2014). The better fit of the ligand present on the microbes (against the receptors present on the host), the stronger the interaction (Goyal et al., 2018; Patin et al., 2019). Fungal ligands are a class of evolutionarily conserved structures called the pathogen associated molecular patterns (PAMPs) and are recognized by receptors present on the host surface called pattern recognition receptors (PRRs). Post internalization, fungi are primarily recognized by the innate cells (e.g., macrophages and dendritic cells) of the immune system (Mogensen, 2009). The main receptors that recognize the fungal-derived PAMPs are Toll-like receptor (TLR like TLR2, TLR4, and TLR9), C-type lectin receptor (CLR like Dectin1 and Dectin2), Nod-like receptor (NLR), Rig-like receptor (RLR), complement receptor, and mannose binding lectin (MBL) (Akira et al., 2006; van de Veerdonk et al., 2008; Hatinguais et al., 2020). These receptors are a crucial component of fungal recognition and trigger an innate immune response.

The host immune response mainly consists of two types, innate and adaptive immunity (Chaplin, 2010; Aristizabal and González, 2013; Netea et al., 2019). Cell-mediated innate immunity is through antigen-presenting cells (APC), which recognize the fungal antigen and process and present it to the T cells. The T cells that participate in antifungal immunity involve Th (helper T cells) cells, Tc cells (cytotoxic T cells), and Treg (regulatory T cells) cells (Hamad et al., 2018). As soon as the body’s immune cells see the foreign fungus, a chain reaction is initiated. Phagocytosis of the fungal pathogen is mediated by neutrophils, macrophages, and dendritic cells, and the oxidative burst kills fungal pathogen by the activity of NADPH oxidase (Rosales and Uribe-Querol, 2017; Warris and Ballou, 2019). The deficiency of this enzyme disrupts the formation of reactive oxygen species (ROS) and makes an individual more susceptible to fungal infection (Hamad et al., 2018). The non-oxidative killing of the fungal pathogen is enhanced by antimicrobial peptides (AMPs) that disrupt the fungal cell wall and also produce neutrophil extracellular traps (NETs) consisting of calprotectin, which induces antifungal activity (Pathakumari et al., 2020; Ulfig and Leichert, 2021). Calprotectin released from NET is an antimicrobial heterodimer that helps in clearing fungus like *Candida*, and its deficiency leads to increased fungal burden (Urban et al., 2009). Innate immune response activates adaptive immunity, which is enhanced by both humoral and cell-mediated immune response, aiding in recognizing fungal antigen, generating inflammation, activating the complement cascade, and further leading to opsonization and neutralization of fungal pathogen (Drummond et al., 2014).

Characterization of single gene defects that predispose individuals to fungal infections needs urgent attention. Monogenic causes for susceptibility of invasive fungal infections have unmasked novel molecules and key signaling pathways in defense against mucosal and systemic antifungal threats (Lionakis et al., 2014; Constantine and Lionakis, 2020). Genetic changes in some key genes play a crucial role in host-pathogen recognition (Kumar et al., 2018; Cunha and Carvalho, 2019; Merkhofer and Klein, 2020). Fungal β-glucan (PAMP) activity can be masked through a change in cell wall components and thus prevent target recognition (Plato et al., 2015). A genetic defect in the different types of PRR families makes the host susceptible to fungal infection (Netea et al., 2012). Defect in the CLR Dectin1, encoded by *CLEC7A* (C-type lectin domain containing 7A) predisposes humans to invasive aspergillosis (IA), chronic mucocutaneous candidiasis (CMC), and recurrent vulvovaginal candidiasis (RVVC) (Reid et al., 2009; Plantinga et al., 2012; Cunha et al., 2018). The *CLEC7A* intronic SNPs rs3901533 and rs7309123 are associated with susceptibility to invasive pulmonary aspergillosis (IPA) in patients with hematologic diseases (Taylor et al., 2007; Sainz et al., 2012). Dectin-1 *Y238X* polymorphism leading to diminished Dectin-1 receptor activity plays a role in RVVC and IA (Plantinga et al., 2009; Cunha et al., 2010; Zahedi et al., 2016). Dectin-1 gene variant also contributes susceptibility to coccidioidomycosis (del Pilar Jiménez-A et al., 2008). Another receptor MBL interacts with pathogens, helps in triggering an immune response, and plays an important role in innate immunity. Deficiency in MBL expression is associated with susceptibility to RVVC (Carvalho et al., 2010) and pneumocystis pneumonia (PCP) (Yanagisawa et al., 2020). Polymorphism in MBL is also associated with chronic cavitary pulmonary aspergillosis and *Candida* infection (Vaid et al., 2007).

SNPs in TLR lead to genetic variation that results in susceptibility to *Candida* and *Aspergillus* infections (Cunha et al., 2010; Table 1). Mutation in *TLR1* is associated with candidemia (Ferwerda et al., 2009; Plantinga et al., 2009, 2012). Genetic variation in the PRR TLR4 can also make an individual susceptible to diseases like IPA (Cunha and Carvalho, 2019). Polymorphism in Asp299Gly and Thr399lle present in the *TLR4* impacts hyporesponsiveness to lipopolysaccharide signaling in epithelial cells or alveolar macrophages and results in chronic cavitary pulmonary aspergillosis (Arbour et al., 2000; Carvalho et al., 2008). In addition, polymorphism in immune response *NOD2* (nucleotide binding oligomerization domain containing 2) gene results in IPA. Variation in another receptor type RLR is also associated with *Candida* infection (Gresnigt et al., 2018; Jaeger et al., 2019). Thus, a mutation in the gene coding for a receptor is an important susceptibility factor for CMC and plays a central role in host immune response (Glocker et al., 2009).

## Genetic Polymorphism of the Immune System Linked to Fungal Infections

Genetic variants leading to immunological susceptibility have long been recognized with a few immunodeficiencies characterized by their vulnerability to IFIs (Pana et al., 2014; Maskarinec et al., 2016; Merkhofer and Klein, 2020). Deficiency in *PTX3* (Pentraxin 3), which is involved in innate immunity, leads to susceptibility toward IA (Garlanda et al., 2002). Recently, downregulation of cluster of differentiation molecules CD50 and CD80 has been shown to make an individual susceptible to *Trichophyton* infection (Hamad et al., 2018). Polymorphism in the *CX3CR1* gene (C-X3-C motif chemokine receptor 1, encoding chemokine receptor) is associated with fungal infection in the gut, and it plays an important role in antifungal activity through activation of Th17 cells and IgG antibody response (Kumar et al., 2018). *Candida* infections (ranging from mucosal to bloodstream, including deep-seated infections) are influenced by genetic variants in the human genomes like polymorphism in signal transducer and activator of transcription protein-coding genes *STAT1* and *STAT3* (Plantinga et al., 2012; Smeekens et al., 2013). The important adaptor protein *CARD9* (caspase recruitment domain-containing protein 9) is involved in signal transduction from a variety of receptors, and mutation in this gene not only leads to mucosal infection but also is associated with IFIs, development of autoimmune diseases, inflammatory bowel disease (IBD), and cancer (Glocker et al., 2009; Drummond et al., 2018). *CARD9* plays an important role in Th17 cell differentiation and helps in the release of cytokines (Vautier et al., 2010; Speakman et al., 2020; Vornholz and Ruland, 2020). Recently, defects in *CARD9* and *STAT3* have been found to cause IFI with gastrointestinal manifestations (Vinh, 2019) and mutation in *STAT3* results in reduced Th17 cells causing candidiasis (Engelhardt and Grimbacher, 2012). A heterozygous missense mutation in *STAT1* is associated with coccidioidomycosis and histoplasmosis (Sampaio et al., 2013). Mutation in another transcription factor GATA2 (GATA-binding factor 2) makes patients vulnerable to myeloid malignancy who have a high risk for treatment-associated IFIs involving aspergillosis and candidiasis (Spinner et al., 2014; Donadieu et al., 2018; Vedula et al., 2021). ZNF341 (zinc finger protein 341) is a transcription factor that resides in the nucleus and regulates the activity of *STAT1* and *STAT3* genes. ZNF341-deficient patients lack Th17 cells and have an excess of Th2 cells and low memory B cells. Upon *Candida* infection, individuals with *STAT3* mutation result in hyper–immunoglobulin E syndrome (HIES) associated with defective Th17 cell differentiation and characterized by elevated serum IgE (Béziat et al., 2018; Frey-Jakobs et al., 2018; Egri et al., 2021). Patients with *AIRE* (autoimmune regulator) gene mutations are also susceptible to *Candida albicans* infection and present themselves with autoimmune disorders (Pedroza et al., 2012; de Albuquerque et al., 2018). Genes encoding immune molecules of the adaptive immune system play an important role in controlling fungal invasion (Kawai and Akira, 2007). Immunoglobulins (Igs) IgG, IgA, IgE, and IgM as part of the humoral adaptive immunity mediate protection through direct actions on fungal cells, and classical mechanisms such as phagocytosis and complement activation are affected in case of mutations in genes coding for those Igs (Kaufman, 1985; Lionakis et al., 2014; Table 1). MHC class II defects lead to primary immunodeficiency disease (PIDD) and make individuals susceptible to a high rate of fungal infection like Candidiasis and PCP (Lanternier et al., 2013; Abd Elaziz et al., 2020). Mutation in *CARD9* and *DOCK8* (dedicator of cytokinesis 8) among PIDD individuals makes them susceptible to *Malassezia* infection, and deficiency in *STAT3* leads to IPA (Abd Elaziz et al., 2020). Summary of the immune-related genes responsible for susceptibility to fungal infection is highlighted in Table 1.

Interleukins (ILs) play a crucial role during fungal infection and help in the maturation of B cells and antibody secretion, which helps fight fungal infections (Antachopoulos and Roilides, 2005; Verma et al., 2015; Sparber and LeibundGut-Landmann, 2019; Griffiths et al., 2021). Mutations in genes encoding for members of the IL-1 family are associated with acute and chronic inflammation and are essential for the innate response to infection (Caffrey et al., 2015; Griffiths et al., 2021). Genetic variation in IL-6 results in blastomycosis (Merkhofer et al., 2019), and defect in IL-10 and IL-6 signaling affects *STAT3*, a key immune response molecule. Genetic variation in IL-10 has also been found to be the underlying cause of susceptibility toward fungal infections like IA (Zaas, 2006). IL-10 mutation makes an individual susceptible to *Candida* and *Coccidiodes immitis* infection (Fierer, 2006), and IL-4 polymorphism resulted in susceptibility toward *Candida* infection (Babula et al., 2005; Choi et al., 2005). SNP in rs2243250, known to influence IL-4 production, is associated with susceptibility to PCP in HIV-positive patients (Wójtowicz et al., 2019). In addition, deficiency of interleukin IL-17 and IL-22 production as a result of genetic mutation has been reported to be the cause of RVVC (Sobel, 2016). IL-2 mutation too predisposes individuals to invasive fungal infection like histoplasmosis by affecting T cell functions (Smeekens et al., 2013; Lionakis et al., 2014; Kumaresan et al., 2017; Pathakumari et al., 2020). The emerging role of the IL-12 family of cytokines in the fight against candidiasis has been reported (Ashman et al., 2011; Thompson and Orr, 2018). IL-12RB1 (interleukin 12 receptor subunit beta 1) impairs the development of human IL-17 producing T cells (Huppler et al., 2012; Johnson et al., 2012; Thompson and Orr, 2018), and mutations inherited might be responsible for histoplasmosis (León-Lara et al., 2020). RAR-related orphan receptor C (*RORC*) encoding transcription factors play an integral role in both IL-17 and IFNγ pathways in CMC (De Luca et al., 2007; Constantine and Lionakis, 2020). Deficiency of tyrosine kinase 2 (Tyk2) that participates in signal transduction for various cytokine receptors leads to impaired helper T cell type 1 (Th1) differentiation and accelerated helper T cell type 2 (Th2) differentiation in candidiasis (Minegishi et al., 2006). Mutation in the main inflammasome NLRP3 (NOD-, LRR-, and pyrin domain-containing protein 3), associated with fungal infection, leads to susceptibility toward RVVC or IPA (Kasper et al., 2018; Wang et al., 2020; Briard et al., 2021). Also, mutations in key recombination activating genes (*RAG1* and *RAG2*) lead to loss of T and B cells, making individuals susceptible to CMC and a broad spectrum of pathogens (Schuetz et al., 2008; Delmonte et al., 2018). Genetic polymorphism in the IL-17 family genes, which encode for the Th17 cellular differentiation, results in an individual’s susceptibility toward fungal infection (Hamad et al., 2018). One of the key signaling molecule pathways, the IL-17R signaling is dependent on Act1 (Actin1—a conserved protein that helps in key cellular processes), and mutation in the gene coding for Actin1 leads to defect in IL-17R signaling pathway, which ultimately fails to provide immunity against CMC (Boisson et al., 2013). IL-17RA binds to homo- and heterodimers of IL-17A and IL-17F, and its deficiency or genetic mutation in any of the gene coding for receptors IL-17RA or IL-17RC leads to CMC (Puel et al., 2011; Sawada et al., 2021).

Mutation in *DOCK8* characterized by elevated IgE level is also known to be responsible for recurrent fungal infections like IA and mucocutaneous candidiasis (Biggs et al., 2017; Nahum, 2017). During *Aspergillus* infection, tumor necrosis factor-alpha (TNFα) enhances the phagocytic activity and the polymorphic site in TNF promotor predisposes individuals to IA (Roilides et al., 1998; Sainz et al., 2007). Neutrophil cytosolic factors (NCFs) are part of the group of proteins that form the enzyme complex called NADPH oxidase, and mutation in any of the key genes *NCF1*, *NCF2*, and *NCF4* leads to impaired fungal eradication (as in aspergillosis) due to non-functional NADPH oxidase (Panday et al., 2015; Giardino et al., 2017; Dinauer, 2019; Wu et al., 2019). Decreased myeloperoxidase (MPO) activity (inability to produce hypochlorous acid) in neutrophils leads to delayed killing of pathogen and makes an individual susceptible to invasive *Candida* infection (Aratani et al., 1999; Merkhofer and Klein, 2020). Myeloperoxidase mutants lead to impaired ROS production, making the host susceptible to infection, and thus, both MPO and NADPH oxidase mutants are unable to eradicate fungal threats like chronic granulomatous disease (CGD) and IA (Lehrer and Cline, 1969; Aratani et al., 2004; Segal and Romani, 2009; Ren et al., 2012). Cytochrome b -245 is a primary component of the microbicidal oxidase system of phagocytes encoded by the alpha and beta chains *CYBA* and *CYBB/*Nox2 (NADPH oxidase 2), respectively (Stasia, 2016), and cytochrome b deficiency is also linked to CGD (Clark, 1999; Stasia et al., 2003; Kutukculer et al., 2019). Recently, it has been reported that mutants in the ferroxidase gene make individuals susceptible to mucormycosis (Navarro-Mendoza et al., 2018), an infection that has been affecting COVID-19 patients (Raut and Huy, 2021). Thus, mutations of key genes of the immune system play an important role in fungal resistance, and interestingly, genetic polymorphisms in these genes have revealed some links with human ancestry.

## Human Ancestry and Genetic Predisposition to Fungal Infections

There is limited research investigating the link between genetic polymorphism in key immune genes, human ancestry, and susceptibility toward fungal infection (Figure 1). But recent research outcomes aided by genomic sequencing point toward an interesting fact. Infection with the fungus *Coccidioides immitis* among Filipino ancestry was found to be common among men and non-white persons causing coccidioidomycosis (van Burik and Magee, 2001). Studies on DNA, which provides genetic information transferred from ancestors to their family members and relatives, indicate that the Hmong ancestry are more susceptible to fungal infection (Xiong et al., 2013). In another report, genetic differentiation among the Hmong ancestry originating from Wisconsin makes them more susceptible to blastomycosis. The Chinese Han population was found to suffer due to poor metabolism as a result of the *CYP2C19* gene (cytochrome P450 2C19) polymorphism involved in the metabolism of xenobiotics. This is one of the direct evidence to prove the role played by genetic polymorphisms in IFIs among a particular human race. Interestingly, polymorphism in the *CYP2C19* allele (because of the presence of variant rs12248560) has been reported to cause aspergillosis among the Chileans (Espinoza et al., 2019). Similarly, deficiency as a result of a mutation in the gene coding for CD40L (binds to CD40 cells and plays role in B cell proliferation) influences susceptibility to PCP among people belonging to the Chinese mainland (Du et al., 2019). It was also reported that genetic variation in *CARD9* led to increased susceptibility toward *Candida* infections in the African population (Rosentul et al., 2012).

SNP plays an important role in fungal infection affecting particular ancestral populations (Hughes et al., 2008; Dominguez-Andres and Netea, 2019). SNPs in genes like *ARNT2* (aryl hydrocarbon receptor nuclear translocator 2) and *CX3CR1* are responsible for cytokine activation, and polymorphism in these genes has been found to play an important role in the invasiveness of aspergillosis infection among European ancestry (Lupiañez et al., 2020). Variations in the PRR MBL and mannose-binding lectin-associated serine protease-2 (MASP-2) proteins were shown to be responsible for sporotrichosis in the Chinese population. It was observed that individuals with elevated levels of the protein are more susceptible to *Sporothrix* infection (Bao et al., 2019). Another importance of SNP is associated with the varying protein expression levels associated with autoimmune diseases (Lionakis, 2012; Jonkers and Wijmenga, 2017). SNPs in cytokine coding genes influence the low production of TNFα, IFNγ, and IL-10, and it was observed that these variations make the Caucasian population susceptible to fungal infections (Larcombe et al., 2005). In a recent study, genetic variant of the key immune adapter MyD88 (myeloid differentiation factor 88) in the Chinese Han population was found to be associated with higher fungal infection and it was shown that the defect in Dectin1 was the primary cause (Chen et al., 2019). Susceptibility to candidiasis and IPA as a result of a defect in Dectin1 was observed in the Dutch family (Ferwerda et al., 2009; Chai et al., 2011). In addition, susceptibility to histoplasmosis as a result of the human leukocyte antigen B22 (*HLA-B22*) variant was reported in the Mexican population (Taylor et al., 1997). The human race thus plays a crucial role in fungal invasion as seen among white transplant recipients who are more susceptible compared to black recipients due to differences in their pharmacogenetics (Boehme et al., 2014). All the above studies show direct links of human ancestry to fungal diseases and indicate how genetic mutations among the human race make them predisposed to certain fungal infections (Table 1).

## Discussion

Fungi play an important role in the human microbiome (Huseyin et al., 2017; Perez et al., 2021; Tiew et al., 2021). In this review, we have focused on genetic predisposition to human fungal infections and discussed the link that exists between ancestry and susceptibility to IFIs. Among those fungi that are commensal with the warm-blooded host, few turn pathogenic under not so well-defined conditions (Hall and Noverr, 2017; Jacobsen, 2019; Limon et al., 2017). Such conversion to pathogenic forms is aided by external factors like environment, immunological status, and most importantly host genetics (Kobayashi, 1996; Kumar et al., 2018; Figure 1). As we learn more about fungal biology, we also understand genetic signatures in the host that make them prone to fungal infections. This is explained by the term genetic predisposition, and external players like the environment also play a role in triggering an autoimmune, inflammatory, or allergic reaction to fungal infections (Figure 1). Identification of fungal allergens has become challenging because most of the allergens mimic immune molecules (Pfavayi et al., 2020). We have seen how mutations in key recognition molecules (Table 1) play a trigger for several fungal infections. We looked into variations introduced by SNPs that are present in the immune response genes (Table 1) critical for fungal infections. The polymorphism in the immune genes (*PTX3, CX3CR1, CARD9*, *STAT3*, and others, Figure 1) make the host susceptible (Garlanda et al., 2002; Kumar et al., 2018; Vinh, 2019), and defect in interleukins (e.g., IL-4, IL-10) leads to genetic predisposition toward fungal infection (Babula et al., 2005; Choi et al., 2005; Zaas, 2006; Table 1). The study of these genes helps us to understand the relationship between genetic polymorphism and the cellular phenotype of host, pathogen, and associated defense mechanisms (Sardinha et al., 2011). Thus, the composition of both host and pathogen plays important role in disease progression, and the challenge is to identify the genetic components involved in pathogenesis.

A few studies point toward a link between human ancestry and genetic predisposition to fungal infections (van Burik and Magee, 2001; Ferwerda et al., 2009; Xiong et al., 2013; Chen et al., 2019; Du et al., 2019; Espinoza et al., 2019; Table 1). Mutations in several components of the immune system make certain human ancestral descendants more prone to fungal infections. Few studies have looked into genetic associations and human ancestry. This aspect is an important and emerging research area in terms of population genetics (Hirschhorn et al., 2002; Gnat et al., 2021). Mutation in key genes relating to the immune system of the host makes certain ancestral descendants susceptible to fungal infections as we observe in the case of certain European, African, and Caucasian individuals (Larcombe et al., 2005; Kwizera et al., 2019; Pfavayi et al., 2020), making them more susceptible to emerging fungal pathogens (Figure 1). Such fungi are a threat to global public health and can colonize the skin, spread from person to person, and cause many high-risk diseases (Lamoth and Kontoyiannis, 2018). To deal with such organisms, we require better surveillance methods, rapid and accurate diagnostics, and decolonization protocols that include administration of antimicrobial or antiseptic agents and new antifungal drugs (Jeffery-Smith et al., 2018; Jackson et al., 2019; Chowdhary et al., 2020; Fisher et al., 2020; Steenwyk et al., 2020). Genome-wide association studies (GWAS) would help us to evaluate the difference in the DNA sequences and understand heritability, disease risk, and susceptibility to antifungals (Bloom et al., 2019; Guo et al., 2020; Figure 1). From genome sequencing, genomic variations like SNPs, variable number tandem repeats (VNTRs), and insertion/deletions (Indels) can be identified. Structural genome variations like aneuploidy and copy number variations (CNVs) also provide important clues to fungal virulence (Tsai and Nelliat, 2019). During fungal microevolution, many of these events like insertion/deletion of genes, loss of heterozygosity (LOH), and genome plasticity help fungus to adapt against antifungal drugs and harsh host environment (Beekman and Ene, 2020). Thus, as part of preventive medicine, a better understanding of host genetics behind fungal infection will help us to study infectious diseases through modern genomic approaches and offer personalized therapy against invasive fungal diseases.

## Author Contributions

SD conceptualized, reviewed, and approved the manuscript. BN drafted the manuscript, revised the article critically, and provided critical suggestions. SA contributed toward artwork and reviewed the manuscript. SL provided critical review and revised intellectual content. All authors contributed to the article and approved the submitted version.

## Conflict of Interest

The authors declare that the research was conducted in the absence of any commercial or financial relationships that could be construed as a potential conflict of interest.

## Publisher’s Note

All claims expressed in this article are solely those of the authors and do not necessarily represent those of their affiliated organizations, or those of the publisher, the editors and the reviewers. Any product that may be evaluated in this article, or claim that may be made by its manufacturer, is not guaranteed or endorsed by the publisher.

## Abbreviations

PRR: Pattern Recognition Receptor
PAMP: Pathogen Associated Molecular Patterns
TLR: Toll-like Receptor
CLR: C-type Lectin Receptor
NLR: Nod-like Receptor
RLR: Rig-like Receptor
Th cells: Helper T cells
Tc cells: Cytotoxic T cells
Treg cells: Regulatory T cells
ILs: Interleukins
Igs: Immunoglobulins
MBL: Mannose Binding Lectin
*CARD9*: Caspase Recruitment Domain-containing protein 9
CD: Cluster of Differentiation
NET: Neutrophil Extracellular Trap
IFI: Invasive Fungal Infection
*PTX3*: Pentraxin3
*CX3CR1*: C-X 3-C Motif Chemokine Receptor 1
Act1: Actin 1
SNPs: Single Nucleotide Polymorphisms
*CYP2C19*: Cytochrome P450 2C19
*ARNT2*: Aryl hydrocarbon Receptor Nuclear Translocator 2
TNFα: Tumor Necrosis Factor-alpha
IFNγ: Interferon-gamma
*MyD88*: Myeloid differentiation primary response 88
*STAT1*: Signal Transducer and Activator of Transcription 1
*STAT3*: Signal Transducer and Activator of Transcription 3
AMP: Anti-Microbial Peptide
APC: Antigen Processing Cell
GWAS: Genome-Wide Association Studies
VNTR: Variable Number Tandem Repeat
Indel: Insertions Deletions
CNV: Copy Number Variation
LOH: Loss of Heterozygosity
MPO: Myeloperoxidase
ROS: Reactive Oxygen Species
CGD: Chronic Granulomatous Disease
CYBB: Cytochrome B beta chain
CYBA: Cytochrome B alpha chain
MASP-2: Mannose-binding lectin-associated serine protease-2
NADPH: Nicotinamide Adenine Dinucleotide Phosphate
NCF: Neutrophil Cytosolic Factor
*CLEC7A*: C-Type Lectin Domain Containing 7A
*NOD2*: Nucleotide-binding Oligomerization Domain containing 2
*RAG*: Recombination Activating Genes
GATA2: GATA-binding factor 2
ZNF341: Zinc Finger Protein 341
IL-12RB1: Interleukin 12 Receptor subunit Beta 1
*AIRE*: Autoimmune Regulator
*RORC*: RAR-related Orphan Receptor C
*DOCK8*: Dedicator of Cytokinesis 8
Tyk2: Tyrosine Kinase 2
CMC: Chronic Mucocutaneous Candidiasis
NLRP3: NOD-, LRR- and Pyrin domain-containing protein 3
PCP: Pneumocystis pneumonia
IPA: Invasive Pulmonary Aspergillosis
HLA-B22: Human Leukocyte Antigen–B22
Nox2: NADPH oxidase 2
PDA: Pancreatic Ductal Adenocarcinoma
IA: Invasive Aspergillosis
HIES: Hyper–Immunoglobulin E Syndrome
RVVC: Recurrent Vulvovaginal Candidiasis
IBD: Inflammatory bowel disease
PIDD: Primary Immunodeficiency disease.

## References

[B1] Abd Elaziz D. Abd El-Ghany M. Meshaal S. El Hawary R. Lotfy S. Galal N. (2020). Fungal infections in primary immunodeficiency diseases. *Clin. Immunol. Orlando Fla* 219:108553. 10.1016/j.clim.2020.108553 32738296

[B2] Akira S. Uematsu S. Takeuchi O. (2006). Pathogen recognition and innate immunity. *Cell* 124 783–801. 10.1016/j.cell.2006.02.015 16497588

[B3] Antachopoulos C. Roilides E. (2005). Cytokines and fungal infections. *Br. J. Haematol.* 129 583–596. 10.1111/j.1365-2141.2005.05498.x 15916680

[B4] Aratani Y. Koyama H. Nyui S. Suzuki K. Kura F. Maeda N. (1999). Severe impairment in early host defense against *Candida albicans* in mice deficient in myeloperoxidase. *Infect. Immun.* 67 1828–1836. 10.1128/IAI.67.4.1828-1836.1999 10085024 PMC96534

[B5] Aratani Y. Kura F. Watanabe H. Akagawa H. Takano Y. Suzuki K. (2004). In vivo role of myeloperoxidase for the host defense. *Jpn. J. Infect. Dis.* 57:S15. 15507755

[B6] Arbour N. C. Lorenz E. Schutte B. C. Zabner J. Kline J. N. Jones M. (2000). TLR4 mutations are associated with endotoxin hyporesponsiveness in humans. *Nat. Genet.* 25 187–191. 10.1038/76048 10835634

[B7] Aristizabal B. González Á (2013). “Innate immune system,” in *Autoimmunity: From Bench to Bedside*, eds Anaya J. M. Shoenfeld Y. Rojas-Villarraga A. Levy R. A. Cervera R. (Bogota: El Rosario University Press). 29087650

[B8] Ashman R. B. Vijayan D. Wells C. A. (2011). IL-12 and related cytokines: function and regulatory implications in *Candida albicans* infection. *Clin. Dev. Immunol.* 2011:686597. 10.1155/2011/686597 21052539 PMC2968417

[B9] Aykut B. Pushalkar S. Chen R. Li Q. Abengozar R. Kim J. I. (2019). The fungal mycobiome promotes pancreatic oncogenesis via activation of MBL. *Nature* 574 264–267. 10.1038/s41586-019-1608-2 31578522 PMC6858566

[B10] Babula O. Lazdâne G. Kroica J. Linhares I. M. Ledger W. J. Witkin S. S. (2005). Frequency of interleukin-4 (IL-4) -589 gene polymorphism and vaginal concentrations of IL-4, nitric oxide, and mannose-binding lectin in women with recurrent vulvovaginal candidiasis. *Clin. Infect. Dis. Off. Publ. Infect. Dis. Soc. Am.* 40 1258–1262. 10.1086/429246 15825027

[B11] Bao F. Fu X. Yu G. Wang Z. Liu H. Zhang F. (2019). Mannose-Binding lectin and mannose-binding lectin-associated serine protease-2 genotypes and serum levels in patients with sporotrichosis. *Am. J. Trop. Med. Hyg.* 101 1322–1324. 10.4269/ajtmh.19-0470 31549610 PMC6896860

[B12] Beekman C. N. Ene I. V. (2020). Short-term evolution strategies for host adaptation and drug escape in human fungal pathogens. *PLoS Pathog.* 16:e1008519. 10.1371/journal.ppat.1008519 32407384 PMC7224449

[B13] Béziat V. Li J. Lin J.-X. Ma C. S. Li P. Bousfiha A. (2018). A recessive form of hyper-IgE syndrome by disruption of ZNF341-dependent STAT3 transcription and activity. *Sci. Immunol.* 3:eaat4956. 10.1126/sciimmunol.aat4956 29907691 PMC6141026

[B14] Biggs C. M. Keles S. Chatila T. A. (2017). DOCK8 deficiency: insights into pathophysiology, clinical features and management. *Clin. Immunol. Orlando Fla* 181 75–82. 10.1016/j.clim.2017.06.003 28625885 PMC5555255

[B15] Blackwell M. (2011). The fungi: 1, 2, 3. 5.1 million species? *Am. J. Bot.* 98 426–438. 10.3732/ajb.1000298 21613136

[B16] Bloom J. S. Boocock J. Treusch S. Sadhu M. J. Day L. Oates-Barker H. (2019). Rare variants contribute disproportionately to quantitative trait variation in yeast. *ELife* 8:e49212. 10.7554/eLife.49212 31647408 PMC6892613

[B17] Boehme A. K. McGwin G. Andes D. R. Lyon G. M. Chiller T. Pappas P. G. (2014). Race and invasive fungal infection in solid organ transplant recipients. *Ethn. Dis.* 24 382–385. 25065083

[B18] Boisson B. Wang C. Pedergnana V. Wu L. Cypowyj S. Rybojad M. (2013). An ACT1 mutation selectively abolishes interleukin-17 responses in humans with chronic mucocutaneous candidiasis. *Immunity* 39 676–686. 10.1016/j.immuni.2013.09.002 24120361 PMC3873857

[B19] Briard B. Malireddi R. K. S. Kanneganti T.-D. (2021). Role of inflammasomes/pyroptosis and PANoptosis during fungal infection. *PLoS Pathog.* 17:e1009358. 10.1371/journal.ppat.1009358 33735255 PMC7971547

[B20] Caffrey A. K. Lehmann M. M. Zickovich J. M. Espinosa V. Shepardson K. M. Watschke C. P. (2015). IL-1α signaling is critical for leukocyte recruitment after pulmonary *Aspergillus fumigatus* challenge. *PLoS Pathog.* 11:e1004625. 10.1371/journal.ppat.1004625 25629406 PMC4309569

[B21] Carris L. M. Little C. R. Stiles C. M. (2012). *Introduction to Fungi. The Plant Health Instructor*. 10.1094/PHI-I-2012-0426-01

[B22] Carvalho A. Cunha C. Pasqualotto A. C. Pitzurra L. Denning D. W. Romani L. (2010). Genetic variability of innate immunity impacts human susceptibility to fungal diseases. *Int. J. Infect. Dis. IJID Off. Publ. Int. Soc. Infect. Dis.* 14 e460–e468. 10.1016/j.ijid.2009.06.028 19828347

[B23] Carvalho A. Pasqualotto A. C. Pitzurra L. Romani L. Denning D. W. Rodrigues F. (2008). Polymorphisms in toll-like receptor genes and susceptibility to pulmonary aspergillosis. *J. Infect. Dis.* 197 618–621. 10.1086/526500 18275280

[B24] Chai L. Y. A. de Boer M. G. J. van der Velden W. J. F. M. Plantinga T. S. van Spriel A. B. Jacobs C. (2011). The Y238X stop codon polymorphism in the human β-glucan receptor dectin-1 and susceptibility to invasive aspergillosis. *J. Infect. Dis.* 203 736–743. 10.1093/infdis/jiq102 21242599 PMC3072717

[B25] Chaplin D. D. (2010). Overview of the immune response. *J. Allergy Clin. Immunol.* 125 S3–S23. 10.1016/j.jaci.2009.12.980 20176265 PMC2923430

[B26] Chen M.-J. Hu R. Jiang X.-Y. Wu Y. He Z.-P. Chen J.-Y. (2019). Dectin-1 rs3901533 and rs7309123 polymorphisms increase susceptibility to pulmonary invasive fungal disease in patients with acute myeloid leukemia from a Chinese Han population. *Curr. Med. Sci.* 39 906–912. 10.1007/s11596-019-2122-3 31845221

[B27] Choi P. Xanthaki D. Rose S. J. Haywood M. Reiser H. Morley B. J. (2005). Linkage analysis of the genetic determinants of T-cell IL-4 secretion, and identification of Flj20274 as a putative candidate gene. *Genes Immun.* 6 290–297. 10.1038/sj.gene.6364192 15815685

[B28] Chowdhary A. Tarai B. Singh A. Sharma A. (2020). Multidrug-Resistant *Candida auris* infections in critically Ill coronavirus disease patients, India, April-July 2020. *Emerg. Infect. Dis.* 26 2694–2696. 10.3201/eid2611.203504 32852265 PMC7588547

[B29] Clark R. A. (1999). Activation of the neutrophil respiratory burst oxidase. *J. Infect. Dis.* 179 S309–S317. 10.1086/513849 10081501

[B30] Coates M. Blanchard S. MacLeod A. S. (2018). Innate antimicrobial immunity in the skin: a protective barrier against bacteria, viruses, and fungi. *PLoS Pathog.* 14:e1007353. 10.1371/journal.ppat.1007353 30522130 PMC6283644

[B31] Constantine G. M. Lionakis M. S. (2020). Recent advances in understanding inherited deficiencies in immunity to infections. *F1000Res.* 9:F1000 Faculty Rev-243. 10.12688/f1000research.22036.1 32308976 PMC7141165

[B32] Cunha C. Carvalho A. (2019). Genetic defects in fungal recognition and susceptibility to invasive pulmonary aspergillosis. *Med. Mycol.* 57 S211–S218. 10.1093/mmy/myy057 30816966

[B33] Cunha C. Di Ianni M. Bozza S. Giovannini G. Zagarella S. Zelante T. (2010). Dectin-1 Y238X polymorphism associates with susceptibility to invasive aspergillosis in hematopoietic transplantation through impairment of both recipient- and donor-dependent mechanisms of antifungal immunity. *Blood* 116 5394–5402. 10.1182/blood-2010-04-279307 20807886

[B34] Cunha D. de O. Leão-Cordeiro J. A. B. Paula H. D. S. C. Ataides F. S. Saddi V. A. (2018). Association between polymorphisms in the genes encoding toll-like receptors and dectin-1 and susceptibility to invasive aspergillosis: a systematic review. *Rev. Soc. Bras. Med. Trop.* 51 725–730. 10.1590/0037-8682-0314-2018 30517524

[B35] de Albuquerque J. A. T. Banerjee P. P. Castoldi A. Ma R. Zurro N. B. Ynoue L. H. (2018). The role of AIRE in the immunity against *Candida albicans* in a model of human macrophages. *Front. Immunol.* 9:567. 10.3389/fimmu.2018.00567 29666621 PMC5875531

[B36] De Luca A. Montagnoli C. Zelante T. Bonifazi P. Bozza S. Moretti S. (2007). Functional yet balanced reactivity to *Candida albicans* requires TRIF, MyD88, and IDO-dependent inhibition of Rorc. *J. Immunol. Baltim. Md 1950* 179 5999–6008. 10.4049/jimmunol.179.9.5999 17947673

[B37] de Pauw B. E. (2011). What are fungal infections? *Mediterr. J. Hematol. Infect. Dis.* 3:e2011001. 10.4084/MJHID.2011.001 21625304 PMC3103258

[B38] del Pilar Jiménez-A M. Viriyakosol S. Walls L. Datta S. K. Kirkland T. Heinsbroek S. E. M. (2008). Susceptibility to *Coccidioides* species in C57BL/6 mice is associated with expression of a truncated splice variant of Dectin-1 (Clec7a). *Genes Immun.* 9 338–348. 10.1038/gene.2008.23 18418396 PMC3681288

[B39] Delmonte O. M. Schuetz C. Notarangelo L. D. (2018). RAG deficiency: two genes, many diseases. *J. Clin. Immunol.* 38 646–655. 10.1007/s10875-018-0537-4 30046960 PMC6643099

[B40] Dinauer M. C. (2019). Insights into the NOX NADPH oxidases using heterologous whole cell assays. *Methods Mol. Biol. Clifton NJ* 1982 139–151. 10.1007/978-1-4939-9424-3_9 31172471

[B41] Dolinoy D. C. Jirtle R. L. (2008). Environmental epigenomics in human health and disease. *Environ. Mol. Mutagen.* 49 4–8. 10.1002/em.20366 18172876

[B42] Dominguez-Andres J. Netea M. G. (2019). Impact of historic migrations and evolutionary processes on human immunity. *Trends Immunol.* 40 1105–1119. 10.1016/j.it.2019.10.001 31786023 PMC7106516

[B43] Donadieu J. Lamant M. Fieschi C. de Fontbrune F. S. Caye A. Ouachee M. (2018). Natural history of GATA2 deficiency in a survey of 79 French and Belgian patients. *Haematologica* 103 1278–1287. 10.3324/haematol.2017.181909 29724903 PMC6068047

[B44] Drummond R. A. Franco L. M. Lionakis M. S. (2018). Human CARD9: a critical molecule of fungal immune surveillance. *Front. Immunol.* 9:1836. 10.3389/fimmu.2018.01836 30127791 PMC6088205

[B45] Drummond R. A. Gaffen S. L. Hise A. G. Brown G. D. (2014). Innate defense against fungal pathogens. *Cold Spring Harb. Perspect. Med.* 5:a019620. 10.1101/cshperspect.a019620 25384766 PMC4426252

[B46] Du X. Tang W. Chen X. Zeng T. Wang Y. Chen Z. (2019). Clinical, genetic and immunological characteristics of 40 Chinese patients with CD40 ligand deficiency. *Scand. J. Immunol.* 90:e12798. 10.1111/sji.12798 31179555

[B47] Duxbury E. M. Day J. P. Maria Vespasiani D. Thüringer Y. Tolosana I. Smith S. C. (2019). Host-pathogen coevolution increases genetic variation in susceptibility to infection. *ELife* 8:e46440. 10.7554/eLife.46440 31038124 PMC6491035

[B48] Eades C. P. Armstrong-James D. P. H. (2019). Invasive fungal infections in the immunocompromised host: mechanistic insights in an era of changing immunotherapeutics. *Med. Mycol.* 57 S307–S317. 10.1093/mmy/myy136 31292657

[B49] Egri N. Esteve-Solé A. Deyà-Martínez À Ortiz de Landazuri I. Vlagea A. García A. P. (2021). Primary immunodeficiency and chronic mucocutaneous candidiasis: pathophysiological, diagnostic, and therapeutic approaches. *Allergol. Immunopathol. (Madr.)* 49 118–127. 10.15586/aei.v49i1.20 33528939

[B50] Engelhardt K. R. Grimbacher B. (2012). Mendelian traits causing susceptibility to mucocutaneous fungal infections in human subjects. *J. Allergy Clin. Immunol.* 129 294–305; quiz 306–307. 10.1016/j.jaci.2011.12.966 22284928

[B51] Espinoza N. Galdames J. Navea D. Farfán M. J. Salas C. (2019). Frequency of the CYP2C19^∗^17 polymorphism in a Chilean population and its effect on voriconazole plasma concentration in immunocompromised children. *Sci. Rep.* 9:8863. 10.1038/s41598-019-45345-2 31222084 PMC6586657

[B52] Ferwerda B. Ferwerda G. Plantinga T. S. Willment J. A. van Spriel A. B. Venselaar H. (2009). Human dectin-1 deficiency and mucocutaneous fungal infections. *N. Engl. J. Med.* 361 1760–1767. 10.1056/NEJMoa0901053 19864674 PMC2773015

[B53] Fierer J. (2006). IL-10 and susceptibility to *Coccidioides* immitis infection. *Trends Microbiol.* 14 426–427. 10.1016/j.tim.2006.07.009 16893649

[B54] Fisher M. C. Gurr S. J. Cuomo C. A. Blehert D. S. Jin H. Stukenbrock E. H. (2020). Threats posed by the fungal kingdom to humans, wildlife, and agriculture. *mBio* 11:e00449-20. 10.1128/mBio.00449-20 32371596 PMC7403777

[B55] Frey-Jakobs S. Hartberger J. M. Fliegauf M. Bossen C. Wehmeyer M. L. Neubauer J. C. (2018). ZNF341 controls STAT3 expression and thereby immunocompetence. *Sci. Immunol.* 3:eaat4941. 10.1126/sciimmunol.aat4941 29907690 PMC6173313

[B56] Garlanda C. Hirsch E. Bozza S. Salustri A. De Acetis M. Nota R. (2002). Non-redundant role of the long pentraxin PTX3 in anti-fungal innate immune response. *Nature* 420 182–186. 10.1038/nature01195 12432394

[B57] Giardino G. Cicalese M. P. Delmonte O. Migliavacca M. Palterer B. Loffredo L. (2017). NADPH oxidase deficiency: a multisystem approach. *Oxid. Med. Cell. Longev.* 2017:4590127. 10.1155/2017/4590127 29430280 PMC5753020

[B58] Glocker E.-O. Hennigs A. Nabavi M. Schäffer A. A. Woellner C. Salzer U. (2009). A homozygous CARD9 mutation in a family with susceptibility to fungal infections. *N. Engl. J. Med.* 361 1727–1735. 10.1056/NEJMoa0810719 19864672 PMC2793117

[B59] Gnat S. Łagowski D. Nowakiewicz A. (2021). Genetic predisposition and its heredity in the context of increased prevalence of dermatophytoses. *Mycopathologia* 186 163–176. 10.1007/s11046-021-00529-1 33523393 PMC8106586

[B60] Goodrich J. K. Davenport E. R. Clark A. G. Ley R. E. (2017). The relationship between the human genome and microbiome comes into view. *Annu. Rev. Genet.* 51 413–433. 10.1146/annurev-genet-110711-155532 28934590 PMC5744868

[B61] Goyal S. Castrillón-Betancur J. C. Klaile E. Slevogt H. (2018). The interaction of human pathogenic fungi with C-Type lectin receptors. *Front. Immunol* 9:1261. 10.3389/fimmu.2018.01261 29915598 PMC5994417

[B62] Gresnigt M. S. Cunha C. Jaeger M. Gonçalves S. M. Malireddi R. K. S. Ammerdorffer A. (2018). Genetic deficiency of NOD2 confers resistance to invasive aspergillosis. *Nat. Commun.* 9:2636. 10.1038/s41467-018-04912-3 29980664 PMC6035256

[B63] Griffiths J. S. Camilli G. Kotowicz N. K. Ho J. Richardson J. P. Naglik J. R. (2021). Role for IL-1 family cytokines in fungal infections. *Front. Microbiol.* 12:633047. 10.3389/fmicb.2021.633047 33643264 PMC7902786

[B64] Guo X. Zhang R. Li Y. Wang Z. Ishchuk O. P. Ahmad K. M. (2020). Understand the genomic diversity and evolution of fungal pathogen *Candida glabrata* by genome-wide analysis of genetic variations. *Methods San Diego Calif.* 176 82–90. 10.1016/j.ymeth.2019.05.002 31059831

[B65] Hall R. A. Noverr M. C. (2017). Fungal interactions with the human host: exploring the spectrum of symbiosis. *Curr. Opin. Microbiol.* 40 58–64. 10.1016/j.mib.2017.10.020 29132066 PMC5733695

[B66] Hamad M. Mohammad M. G. Abu-Elteen K. H. (2018). Immunity to human fungal infections,” in *Fungi Biology and Applications*, 3rd Edn, 275–298. 10.1002/9781119374312.ch11

[B67] Hatinguais R. Willment J. A. Brown G. D. (2020). PAMPs of the fungal cell wall and mammalian PRRs. *Curr. Top. Microbiol. Immunol.* 425 187–223. 10.1007/82_2020_201 32180018

[B68] Hawksworth D. L. Rossman A. Y. (1997). Where are all the undescribed fungi? *Phytopathology* 87 888–891. 10.1094/PHYTO.1997.87.9.888 18945058

[B69] Hirschhorn J. N. Lohmueller K. Byrne E. Hirschhorn K. (2002). A comprehensive review of genetic association studies. *Genet. Med. Off. J. Am. Coll. Med. Genet.* 4 45–61. 10.1097/00125817-200203000-00002 11882781

[B70] Hughes A. L. Welch R. Puri V. Matthews C. Haque K. Chanock S. J. (2008). Genome-wide SNP typing reveals signatures of population history. *Genomics* 92 1–8. 10.1016/j.ygeno.2008.03.005 18485661 PMC3421839

[B71] Huppler A. R. Bishu S. Gaffen S. L. (2012). Mucocutaneous candidiasis: the IL-17 pathway and implications for targeted immunotherapy. *Arthritis Res. Ther.* 14:217. 10.1186/ar3893 22838497 PMC3580547

[B72] Huseyin C. E. Rubio R. C. O’Sullivan O. Cotter P. D. Scanlan P. D. (2017). The fungal frontier: a comparative analysis of methods used in the study of the human gut mycobiome. *Front. Microbiol.* 8:1432. 10.3389/fmicb.2017.01432 28824566 PMC5534473

[B73] Ibrahim A. S. Voelz K. (2017). The mucormycete-host interface. *Curr. Opin. Microbiol.* 40 40–45. 10.1016/j.mib.2017.10.010 29107938 PMC5733727

[B74] Iliev I. D. Leonardi I. (2017). Fungal dysbiosis: immunity and interactions at mucosal barriers. *Nat. Rev. Immunol.* 17 635–646. 10.1038/nri.2017.55 28604735 PMC5724762

[B75] Jackson B. R. Chow N. Forsberg K. Litvintseva A. P. Lockhart S. R. Welsh R. (2019). On the origins of a species: what might explain the rise of *Candida auris*? *J. Fungi Basel Switz.* 5:E58. 10.3390/jof5030058 31284576 PMC6787658

[B76] Jacobsen I. D. (2019). Fungal infection strategies. *Virulence* 10 835–838. 10.1080/21505594.2019.1682248 31724461 PMC8647851

[B77] Jaeger M. Matzaraki V. Aguirre-Gamboa R. Gresnigt M. S. Chu X. Johnson M. D. (2019). A genome-wide functional genomics approach identifies susceptibility pathways to fungal bloodstream infection in humans. *J. Infect. Dis.* 220 862–872. 10.1093/infdis/jiz206 31241743 PMC6667794

[B78] Jeffery-Smith A. Taori S. K. Schelenz S. Jeffery K. Johnson E. M. Borman A. (2018). *Candida auris*: a review of the literature. *Clin. Microbiol. Rev.* 31:e00029-17. 10.1128/CMR.00029-17 29142078 PMC5740969

[B79] Johnson M. D. Plantinga T. S. van de Vosse E. Velez Edwards D. R. Smith P. B. Alexander B. D. (2012). Cytokine gene polymorphisms and the outcome of invasive candidiasis: a prospective cohort study. *Clin. Infect. Dis. Off. Publ. Infect. Dis. Soc. Am.* 54 502–510. 10.1093/cid/cir827 22144535 PMC3269308

[B80] Jonkers I. H. Wijmenga C. (2017). Context-specific effects of genetic variants associated with autoimmune disease. *Hum. Mol. Genet.* 26 R185–R192. 10.1093/hmg/ddx254 28977443 PMC5886469

[B81] Kasper L. König A. Koenig P.-A. Gresnigt M. S. Westman J. Drummond R. A. (2018). The fungal peptide toxin Candidalysin activates the NLRP3 inflammasome and causes cytolysis in mononuclear phagocytes. *Nat. Commun.* 9:4260. 10.1038/s41467-018-06607-1 30323213 PMC6189146

[B82] Kaufman L. (1985). “The role of specific antibodies of different immunoglobulin classes in the rapid diagnosis of systemic mycotic infections,” in *Rapid Methods and Automation in Microbiology and Immunology*, ed. Habermehl K. O. (Berlin: Springer). 10.1007/978-3-642-69943-6_21

[B83] Kawai T. Akira S. (2007). TLR signaling. *Semin. Immunol.* 19 24–32. 10.1016/j.smim.2006.12.004 17275323

[B84] Kobayashi G. S. (1996). “Disease mechanisms of fungi,” in *Medical Microbiology*, ed. Baron S. 4th Edn, (Galveston, TX: University of Texas). Medical Branch at Galveston. 21413297

[B85] Kruger W. Vielreicher S. Kapitan M. Jacobsen I. D. Niemiec M. J. (2019). Fungal-Bacterial interactions in health and disease. *Pathog. Basel Switz.* 8:E70. 10.3390/pathogens8020070 31117285 PMC6630686

[B86] Kumar V. van de Veerdonk F. L. Netea M. G. (2018). Antifungal immune responses: emerging host-pathogen interactions and translational implications. *Genome Med.* 10:39. 10.1186/s13073-018-0553-2 29801518 PMC5968547

[B87] Kumaresan P. R. da Silva T. A. Kontoyiannis D. P. (2017). Methods of controlling invasive fungal infections using CD8+ T cells. *Front. Immunol.* 8:1939. 10.3389/fimmu.2017.01939 29358941 PMC5766637

[B88] Kutukculer N. Aykut A. Karaca N. E. Durmaz A. Aksu G. Genel F. (2019). Chronic granulamatous disease: two decades of experience from a paediatric immunology unit in a country with high rate of consangineous marriages. *Scand. J. Immunol.* 89:e12737. 10.1111/sji.12737 30506560

[B89] Kwizera R. Musaazi J. Meya D. B. Worodria W. Bwanga F. Kajumbula H. (2019). Burden of fungal asthma in Africa: a systematic review and meta-analysis. *PLoS One* 14:e0216568. 10.1371/journal.pone.0216568 31095641 PMC6521988

[B90] Lamoth F. Kontoyiannis D. P. (2018). The *Candida auris* alert: facts and perspectives. *J. Infect. Dis.* 217 516–520. 10.1093/infdis/jix597 29390110

[B91] Lanternier F. Cypowyj S. Picard C. Bustamante J. Lortholary O. Casanova J.-L. (2013). Primary immunodeficiencies underlying fungal infections. *Curr. Opin. Pediatr.* 25 736–747. 10.1097/MOP.0000000000000031 24240293 PMC4098727

[B92] Larcombe L. Rempel J. D. Dembinski I. Tinckam K. Rigatto C. Nickerson P. (2005). Differential cytokine genotype frequencies among Canadian aboriginal and Caucasian populations. *Genes Immun.* 6 140–144. 15674369 10.1038/sj.gene.6364157

[B93] Lehrer R. I. Cline M. J. (1969). Leukocyte myeloperoxidase deficiency and disseminated candidiasis: the role of myeloperoxidase in resistance to *Candida* infection. *J. Clin. Invest.* 48 1478–1488. 10.1172/JCI106114 5796360 PMC322375

[B94] León-Lara X. Hernández-Nieto L. Zamora C. V. Rodríguez-D’Cid R. Gutiérrez M. E. C. Espinosa-Padilla S. (2020). Disseminated infectious disease caused by histoplasma capsulatum in an adult patient as first manifestation of inherited IL-12Rβ1 deficiency. *J. Clin. Immunol.* 40 1051–1054. 10.1007/s10875-020-00828-0 32710397

[B95] Limon J. J. Skalski J. H. Underhill D. M. (2017). Commensal fungi in health and disease. *Cell Host & Microbe* 22 156–165. 10.1016/j.chom.2017.07.002 28799901 PMC5573128

[B96] Lionakis M. S. (2012). Genetic susceptibility to fungal infections in humans. *Curr. Fungal Infect. Rep.* 6 11–22. 10.1007/s12281-011-0076-4 23087779 PMC3475324

[B97] Lionakis M. S. Netea M. G. Holland S. M. (2014). Mendelian genetics of human susceptibility to fungal infection. *Cold Spring Harb. Perspect. Med.* 4:a019638. 10.1101/cshperspect.a019638 24890837 PMC4031953

[B98] Low C.-Y. Rotstein C. (2011). Emerging fungal infections in immunocompromised patients. *F1000 Med. Rep.* 3:14. 10.3410/M3-14 21876720 PMC3155160

[B99] Lupiañez C. B. Martínez-Bueno M. Sánchez-Maldonado J. M. Badiola J. Cunha C. Springer J. (2020). Polymorphisms within the ARNT2 and CX3CR1 genes are associated with the risk of developing invasive aspergillosis. *Infect. Immun.* 88 e882–e819. 10.1128/IAI.00882-19 31964743 PMC7093133

[B100] Martin E. M. Fry R. C. (2018). Environmental influences on the epigenome: exposure- associated DNA methylation in human populations. *Annu. Rev. Public Health* 39 309–333. 10.1146/annurev-publhealth-040617-014629 29328878

[B101] Maskarinec S. A. Johnson M. D. Perfect J. R. (2016). Genetic susceptibility to fungal infections: what is in the genes? *Curr. Clin. Microbiol. Rep.* 3 81–91. 10.1007/s40588-016-0037-3 27547700 PMC4988683

[B102] Merkhofer R. M. Klein B. S. (2020). Advances in understanding human genetic variations that influence innate immunity to fungi. *Front. Cell. Infect. Microbiol.* 10:69. 10.3389/fcimb.2020.00069 32185141 PMC7058545

[B103] Merkhofer R. M. O’Neill M. B. Xiong D. Hernandez-Santos N. Dobson H. Fites J. S. (2019). Investigation of genetic susceptibility to blastomycosis reveals interleukin-6 as a potential susceptibility locus. *mBio* 10:e01224-19. 10.1128/mBio.01224-19 31213563 PMC6581865

[B104] Minegishi Y. Saito M. Morio T. Watanabe K. Agematsu K. Tsuchiya S. (2006). Human tyrosine kinase 2 deficiency reveals its requisite roles in multiple cytokine signals involved in innate and acquired immunity. *Immunity* 25 745–755. 10.1016/j.immuni.2006.09.009 17088085

[B105] Mogensen T. H. (2009). Pathogen recognition and inflammatory signaling in innate immune defenses. *Clin. Microbiol. Rev.* 22 240–273. 10.1128/CMR.00046-08 Table of Contents 19366914 PMC2668232

[B106] Nahum A. (2017). Chronic mucocutaneous candidiasis: a spectrum of genetic disorders. *LymphoSign J.* 4 87–99.

[B107] Naranjo-Ortiz M. A. Gabaldón T. (2019). Fungal evolution: major ecological adaptations and evolutionary transitions. *Biol. Rev. Camb. Philos. Soc.* 94 1443–1476. 10.1111/brv.12510 31021528 PMC6850671

[B108] Navarro-Mendoza M. I. Pérez-Arques C. Murcia L. Martínez-García P. Lax C. Sanchis M. (2018). Components of a new gene family of ferroxidases involved in virulence are functionally specialized in fungal dimorphism. *Sci. Rep.* 8:7660. 10.1038/s41598-018-26051-x 29769603 PMC5955967

[B109] Netea M. G. Schlitzer A. Placek K. Joosten L. A. B. Schultze J. L. (2019). Innate and adaptive immune memory: an evolutionary continuum in the host’s response to pathogens. *Cell Host Microbe* 25 13–26. 10.1016/j.chom.2018.12.006 30629914

[B110] Netea M. G. Wijmenga C. O’Neill L. A. J. (2012). Genetic variation in Toll-like receptors and disease susceptibility. *Nat. Immunol.* 13 535–542. 10.1038/ni.2284 22610250

[B111] Pana Z.-D. Farmaki E. Roilides E. (2014). Host genetics and opportunistic fungal infections. *Clin. Microbiol. Infect. Off. Publ. Eur. Soc. Clin. Microbiol. Infect. Dis.* 20 1254–1264. 10.1111/1469-0691.12800 25274142

[B112] Panday A. Sahoo M. K. Osorio D. Batra S. (2015). NADPH oxidases: an overview from structure to innate immunity-associated pathologies. *Cell. Mol. Immunol.* 12 5–23. 10.1038/cmi.2014.89 25263488 PMC4654378

[B113] Pathakumari B. Liang G. Liu W. (2020). Immune defence to invasive fungal infections: a comprehensive review. *Biomed. Pharmacother. Biomed. Pharmacother.* 130:110550. 10.1016/j.biopha.2020.110550 32739740

[B114] Patin E. C. Thompson A. Orr S. J. (2019). Pattern recognition receptors in fungal immunity. *In Semin. Cell Dev. Biol.* 89 4–33. 10.1016/j.semcdb.2018.03.003 PMC6461132 29522806

[B115] Pedroza L. A. Kumar V. Sanborn K. B. Mace E. M. Niinikoski H. Nadeau K. (2012). Autoimmune regulator (AIRE) contributes to Dectin-1-induced TNF-α production and complexes with caspase recruitment domain-containing protein 9 (CARD9), spleen tyrosine kinase (Syk), and Dectin-1. *J. Allergy Clin. Immunol.* 129 464–472, 472.e1–3. 21962774 10.1016/j.jaci.2011.08.027

[B116] Perez N. B. Wright F. Vorderstrasse A. (2021). A microbial relationship between irritable bowel syndrome and depressive symptoms. *Biol. Res. Nurs.* 23 50–64. 10.1177/1099800420940787 32705884

[B117] Pfavayi L. T. Sibanda E. N. Mutapi F. (2020). The pathogenesis of fungal-related diseases and allergies in the African population: the state of the evidence and knowledge gaps. *Int. Arch. Allergy Immunol.* 181 257–269. 10.1159/000506009 32069461

[B118] Plantinga T. S. Johnson M. D. Scott W. K. Joosten L. A. B. van der Meer J. W. M. Perfect J. R. (2012). Human genetic susceptibility to *Candida* infections. *Med. Mycol.* 50 785–794. 10.3109/13693786.2012.690902 22662758 PMC5826648

[B119] Plantinga T. S. van der Velden W. J. F. M. Ferwerda B. van Spriel A. B. Adema G. Feuth T. (2009). Early stop polymorphism in human DECTIN-1 is associated with increased candida colonization in hematopoietic stem cell transplant recipients. *Clin. Infect. Dis.* 49 724–732. 10.1086/604714 19614557

[B120] Plato A. Hardison S. E. Brown G. D. (2015). Pattern recognition receptors in antifungal immunity. *Semin. Immunopathol.* 37 97–106. 10.1007/s00281-014-0462-4 25420452 PMC4326652

[B121] Puel A. Cypowyj S. Bustamante J. Wright J. F. Liu L. Lim H. K. (2011). Chronic mucocutaneous candidiasis in humans with inborn errors of interleukin-17 immunity. *Science* 332 65–68. 10.1126/science.1200439 21350122 PMC3070042

[B122] Rai L. S. Wijlick L. V. Bougnoux M. E. Bachellier-Bassi S. d’Enfert C. (2021). Regulators of commensal and pathogenic life-styles of an opportunistic fungus–Candida albicans. *Yeast* 38 243–250. 10.1002/yea.3550 33533498

[B123] Raut A. Huy N. T. (2021). Rising incidence of mucormycosis in patients with COVID-19: another challenge for India amidst the second wave? *Lancet Respir. Med.* 3 265–264. 10.1016/S2213-2600(21)00265-4 PMC8175046 34090607

[B124] Reid D. M. Gow N. A. R. Brown G. D. (2009). Pattern recognition: recent insights from Dectin-1. *Curr. Opin. Immunol.* 21 30–37. 10.1016/j.coi.2009.01.003 19223162 PMC2684021

[B125] Ren R. Fedoriw Y. Willis M. (2012). The molecular pathophysiology, differential diagnosis, and treatment of MPO deficiency. *J. Clin. Exp. Pathol.* 2 2161–2681.

[B126] Richmond J. M. Harris J. E. (2014). Immunology and skin in health and disease. *Cold Spring Harb. Perspect. Med.* 4:a015339. 10.1101/cshperspect.a015339 25452424 PMC4292093

[B127] Roilides E. Dimitriadou-Georgiadou A. Sein T. Kadiltsoglou I. Walsh T. J. (1998). Tumor necrosis factor alpha enhances antifungal activities of polymorphonuclear and mononuclear phagocytes against *Aspergillus fumigatus*. *Infect. Immun.* 66 5999–6003. 10.1128/IAI.66.12.5999-6003.1998 9826384 PMC108760

[B128] Rosales C. Uribe-Querol E. (2017). Phagocytosis: a fundamental process in immunity. *BioMed Res. Int.* 2017:9042851. 10.1155/2017/9042851 28691037 PMC5485277

[B129] Rosentul D. C. Plantinga T. S. Scott W. K. Alexander B. D. van de Geer N. M. D. Perfect J. R. (2012). The impact of caspase-12 on susceptibility to candidemia. *Eur. J. Clin. Microbiol. Infect. Dis.* 31 277–280. 10.1007/s10096-011-1307-x 21706251 PMC3274675

[B130] Sainz J. Lupiáñez C. B. Segura-Catena J. Vazquez L. Ríos R. Oyonarte S. (2012). Dectin-1 and DC-SIGN polymorphisms associated with invasive pulmonary *Aspergillosis* infection. *PLoS One* 7:e32273. 10.1371/journal.pone.0032273 22384201 PMC3288082

[B131] Sainz J. Pérez E. Hassan L. Moratalla A. Romero A. Collado M. D. (2007). Variable number of tandem repeats of TNF receptor type 2 promoter as genetic biomarker of susceptibility to develop invasive pulmonary *Aspergillosis*. *Hum. Immunol.* 68 41–50. 10.1016/j.humimm.2006.10.011 17207711

[B132] Salazar F. Brown G. D. (2018). Antifungal innate immunity: a perspective from the last 10 years. *J. Innate Immun.* 10 373–397. 10.1159/000488539 29768268 PMC6784043

[B133] Sampaio E. P. Hsu A. P. Pechacek J. Bax H. I. Dias D. L. Paulson M. L. (2013). Signal transducer and activator of transcription 1 (STAT1) gain-of-function mutations and disseminated coccidioidomycosis and histoplasmosis. *J. Allergy Clin. Immunol.* 131 1624–1634. 10.1016/j.jaci.2013.01.052 23541320 PMC3746066

[B134] Sardinha J. F. J. Tarlé R. G. Fava V. M. Francio A. S. Ramos G. B. Ferreira L. C. (2011). Genetic risk factors for human susceptibility to infections of relevance in dermatology. *An. Bras. Dermatol.* 86 708–715. 10.1590/s0365-05962011000400013 21987137

[B135] Sawada Y. Setoyama A. Sakuragi Y. Saito-Sasaki N. Yoshioka H. Nakamura M. (2021). The role of IL-17-Producing cells in cutaneous fungal infections. *Int. J. Mol. Sci.* 22:5794. 10.3390/ijms22115794 34071562 PMC8198319

[B136] Schuetz C. Huck K. Gudowius S. Megahed M. Feyen O. Hubner B. (2008). An immunodeficiency disease with RAG mutations and granulomas. *N. Engl. J. Med.* 358 2030–2038. 10.1056/NEJMoa073966 18463379

[B137] Segal B. H. Romani L. R. (2009). Invasive aspergillosis in chronic granulomatous disease. *Med. Mycol.* 47(Suppl. 1) S282–S290. 10.1080/13693780902736620 19296367

[B138] Smeekens S. P. van de Veerdonk F. L. Kullberg B. J. Netea M. G. (2013). Genetic susceptibility to *Candida* infections. *EMBO Mol. Med.* 5 805–813. 10.1002/emmm.201201678 23629947 PMC3779444

[B139] Sobel J. D. (2016). Recurrent vulvovaginal candidiasis. *Am. J. Obstet. Gynecol.* 214 15–21. 10.1016/j.ajog.2015.06.067 26164695

[B140] Sparber F. LeibundGut-Landmann S. (2019). Interleukin-17 in antifungal immunity. *Pathog. Basel Switz.* 8:E54. 10.3390/pathogens8020054 31013616 PMC6630750

[B141] Speakman E. A. Dambuza I. M. Salazar F. Brown G. D. (2020). T cell antifungal immunity and the role of C-Type lectin receptors. *Trends Immunol.* 41 61–76. 10.1016/j.it.2019.11.007 31813764 PMC7427322

[B142] Spinner M. A. Sanchez L. A. Hsu A. P. Shaw P. A. Zerbe C. S. Calvo K. R. (2014). GATA2 deficiency: a protean disorder of hematopoiesis, lymphatics, and immunity. *Blood* 123 809–821. 10.1182/blood-2013-07-515528 24227816 PMC3916876

[B143] Stasia M. J. (2016). CYBA encoding p22(phox), the cytochrome b558 alpha polypeptide: gene structure, expression, role and physiopathology. *Gene* 586 27–35. 10.1016/j.gene.2016.03.050 27048830 PMC5637546

[B144] Stasia M. J. Brion J.-P. Boutonnat J. Morel F. (2003). Severe clinical forms of cytochrome b-negative chronic granulomatous disease (X91-) in 3 brothers with a point mutation in the promoter region of CYBB. *J. Infect. Dis.* 188 1593–1604. 10.1086/379035 14624387

[B145] Steenwyk J. L. Lind A. L. Ries L. N. A. Dos Reis T. F. Silva L. P. Almeida F. (2020). Pathogenic allodiploid hybrids of *Aspergillus* fungi. *Curr. Biol. CB* 30 2495–2507.e7. 32502407 10.1016/j.cub.2020.04.071 PMC7343619

[B146] Taylor M. L. Pérez-Mejía A. Yamamoto-Furusho J. K. Granados J. (1997). Immunologic, genetic and social human risk factors associated to histoplasmosis: studies in the State of Guerrero, Mexico. *Mycopathologia* 138 137–142. 10.1023/a:1006847630347 9468664

[B147] Taylor P. R. Tsoni S. V. Willment J. A. Dennehy K. M. Rosas M. Findon H. (2007). Dectin-1 is required for beta-glucan recognition and control of fungal infection. *Nat. Immunol.* 8 31–38. 10.1038/ni1408 17159984 PMC1888731

[B148] Thompson A. Orr S. J. (2018). Emerging IL-12 family cytokines in the fight against fungal infections. *Cytokine* 111 398–407. 10.1016/j.cyto.2018.05.019 29793796 PMC6299256

[B149] Tiew P. Y. Jaggi T. K. Chan L. L. Y. Chotirmall S. H. (2021). The airway microbiome in COPD, bronchiectasis and bronchiectasis-COPD overlap. *Clin. Respir. J.* 15 123–133. 10.1111/crj.13294 33063421

[B150] Tsai H.-J. Nelliat A. (2019). A Double-Edged sword: aneuploidy is a prevalent strategy in fungal adaptation. *Genes* 10:E787. 10.3390/genes10100787 31658789 PMC6826469

[B151] Ulfig A. Leichert L. I. (2021). The effects of neutrophil-generated hypochlorous acid and other hypohalous acids on host and pathogens. *Cell. Mol. Life Sci.* 78 385–414. 10.1007/s00018-020-03591-y 32661559 PMC7873122

[B152] Underhill D. M. Pearlman E. (2015). Immune interactions with pathogenic and commensal fungi: a two-way street. *Immunity* 43 845–858. 10.1016/j.immuni.2015.10.023 26588778 PMC4865256

[B153] Urban C. F. Ermert D. Schmid M. Abu-Abed U. Goosmann C. Nacken W. (2009). Neutrophil extracellular traps contain calprotectin, a cytosolic protein complex involved in host defense against *Candida albicans*. *PLoS Pathog.* 5:e1000639. 10.1371/journal.ppat.1000639 19876394 PMC2763347

[B154] Vaid M. Kaur S. Sambatakou H. Madan T. Denning D. W. Sarma P. U. (2007). Distinct alleles of mannose-binding lectin (MBL) and surfactant proteins A (SP-A) in patients with chronic cavitary pulmonary aspergillosis and allergic bronchopulmonary aspergillosis. *Clin. Chem. Lab. Med.* 45 183–186. 10.1515/CCLM.2007.033 17311505

[B155] van Burik J. A. Magee P. T. (2001). Aspects of fungal pathogenesis in humans. *Annu. Rev. Microbiol.* 55 743–772. 10.1146/annurev.micro.55.1.743 11544373

[B156] van de Veerdonk F. L. Kullberg B. J. van der Meer J. W. Gow N. A. Netea M. G. (2008). Host-microbe interactions: innate pattern recognition of fungal pathogens. *Curr. Opin. Microbiol.* 11 305–312. 10.1016/j.mib.2008.06.002 18602019

[B157] Vautier S. Sousa M. da G. Brown G. D. (2010). C-type lectins, fungi and Th17 responses. *Cytokine Growth Factor Rev.* 21 405–412. 10.1016/j.cytogfr.2010.10.001 21075040 PMC3001956

[B158] Vedula R. S. Cheng M. P. Ronayne C. E. Farmakiotis D. Ho V. T. Koo S. (2021). Somatic GATA2 mutations define a subgroup of myeloid malignancy patients at high risk for invasive fungal disease. *Blood Adv.* 5 54–60. 10.1182/bloodadvances.2020002854 33570623 PMC7805332

[B159] Verma A. Wüthrich M. Deepe G. Klein B. (2015). Adaptive immunity to fungi. *Cold Spring Harb. Perspect. Med.* 5:a019612. 10.1101/cshperspect.a019612 25377140 PMC4355251

[B160] Vijaya Chandra S. H. Srinivas R. Dawson T. L. Jr. Common J. E. (2021). Cutaneous *Malassezia*: commensal, pathogen, or protector? *Front. Cell. Infect. Microbiol.* 10:614446. 10.3389/fcimb.2020.614446 33575223 PMC7870721

[B161] Vinh D. C. (2019). The molecular immunology of human susceptibility to fungal diseases: lessons from single gene defects of immunity. *Expert Rev. Clin. Immunol.* 15 461–486. 10.1080/1744666X.2019.1584038 30773066

[B162] Vornholz L. Ruland J. (2020). Physiological and pathological functions of CARD9 signaling in the innate immune system. *Curr. Top. Microbiol. Immunol.* 429 177–203. 10.1007/82_2020_211 32415389

[B163] Wang Z. Zhang S. Xiao Y. Zhang W. Wu S. Qin T. (2020). NLRP3 inflammasome and inflammatory diseases. *Oxid. Med. Cell. Longev.* 2020:4063562. 10.1155/2020/4063562 32148650 PMC7049400

[B164] Warris A. Ballou E. R. (2019). Oxidative responses and fungal infection biology. *Semin. Cell Dev. Biol.* 89 34–46. 10.1016/j.semcdb.2018.03.004 29522807

[B165] Wójtowicz A. Bibert S. Taffé P. Bernasconi E. Furrer H. Günthard H. F. (2019). IL-4 polymorphism influences susceptibility to *Pneumocystis jirovecii* pneumonia in HIV-positive patients. *AIDS* 33 1719–1727. 10.1097/QAD.0000000000002283 31225812 PMC6686957

[B166] Wu S.-Y. Weng C.-L. Jheng M.-J. Kan H.-W. Hsieh S.-T. Liu F.-T. (2019). Candida albicans triggers NADPH oxidase-independent neutrophil extracellular traps through dectin-2. *PLoS Pathog.* 15:e1008096. 10.1371/journal.ppat.1008096 31693704 PMC6834254

[B167] Xiong D. Meece J. K. Pepperell C. S. (2013). Genetic research with hmong-ancestry populations: lessons from the literature and a pilot study. *Hmong Stud. J.* 14 1–28.

[B168] Yanagisawa K. Wichukchinda N. Tsuchiya N. Yasunami M. Rojanawiwat A. Tanaka H. (2020). Deficiency of mannose-binding lectin is a risk of *Pneumocystis jirovecii* pneumonia in a natural history cohort of people living with HIV/AIDS in Northern Thailand. *PLoS One* 15:e0242438. 10.1371/journal.pone.0242438 33362211 PMC7757797

[B169] Zaas A. K. (2006). Host genetics affect susceptibility to invasive aspergillosis. *Med. Mycol.* 44 S55–S60. 10.1080/13693780600865481 30408935

[B170] Zahedi N. Abedian Kenari S. Mohseni S. Aslani N. Ansari S. Badali H. (2016). Is human Dectin-1 Y238X gene polymorphism related to susceptibility to recurrent vulvovaginal candidiasis? *Curr. Med. Mycol.* 2 15–19. 10.18869/acadpub.cmm.2.3.15 28681024 PMC5490285

